# T195. GAMMA SYNCHRONY IS DYSFUNCTIONAL DURING COGNITIVE PROCESSING IN FIRST ONSET SCHIZOPHRENIA

**DOI:** 10.1093/schbul/sby016.471

**Published:** 2018-04-01

**Authors:** Anthony Harris, Annie Brennan, Leanne Williams

**Affiliations:** 1 University of Sydney; 2 Stanford University

## Abstract

**Background:**

Schizophrenia is characterised by marked cognitive deficits that are central to the disability caused by the disorder. These deficits are present prior to the onset of schizophrenia, are severe by the time of first presentation and persist. They are thought to arise from a core problem in neural connectivity. Cortical connectivity can be measured using changes in the synchronous activity of the brain in the gamma band (30-100Hz) of the electroencephalogram. This study characterizes the profile of gamma synchrony and behavioural performance during higher-order cognitive processing in schizophrenia.

**Methods:**

Gamma synchrony was acquired during a Continuous Performance Test (CPT) in 59 young people with First Onset Schizophrenia (FOS) and 59 matched controls We quantitated synchrony for regions associated with the fronto-parietal attention and visual networks. Groups were compared on gamma synchrony for intrinsic (pre-stimulus), task-evoked change (relative to baseline) and absolute (not relative to baseline) measures. The relationship between gamma synchrony, CPT accuracy, psychopathology and functioning was also explored.

**Results:**

FOS showed a reduced ability to modulate task-evoked changes in gamma synchrony, in the context of generally higher intrinsic and absolute synchrony, particularly in frontal regions. These gamma synchrony abnormalities in FOS were associated with performance on the CPT, but not with symptoms or functioning.

**Discussion:**

This study is the first to demonstrate that gamma synchrony abnormalities are not limited to perceptual or lower-order cognitive processing. Task-relevant changes in synchrony were constrained by an overall excess of intrinsic background synchrony that was unrelated or not amenable to modulation by specific task demands. This excess in regional synchrony disturbed functional connectivity in central executive circuits and affected cognitive performance but not symptomatically. The results are consistent with gamma synchrony being a measure of dysconnectivity, a core abnormality in schizophrenia.

